# Systematic review and mixed treatment comparison: dressings to heal diabetic foot ulcers

**DOI:** 10.1007/s00125-012-2558-5

**Published:** 2012-04-29

**Authors:** J. C. Dumville, M. O. Soares, S. O’Meara, N. Cullum

**Affiliations:** 1 Department of Health Sciences, University of York, Area 2, Seebohm Rowntree Building, York, YO10 5DD UK; 2 Centre for Health Economics, University of York, York, UK; 3 School of Nursing, Midwifery and Social Work, University of Manchester, Manchester, UK

**Keywords:** Diabetic foot ulcers, Dressings, GRADE, Meta-analysis, Mixed treatment comparison, Systematic review

## Abstract

**Aims/hypothesis:**

Foot ulcers in people with diabetes are a common and serious global health issue. Dressings form a key part of ulcer treatment. Existing systematic reviews are limited by the lack of head-to-head comparisons of alternative dressings in a field where there are several different dressing options. We aimed to determine the relative effects of alternative wound dressings on the healing of diabetic foot ulcers.

**Methods:**

This study was a systematic review involving Bayesian mixed treatment comparison. We included randomised controlled trials evaluating the effects on diabetic foot ulcer healing of one or more wound dressings. There were no restrictions based on language or publication status.

**Results:**

Fifteen eligible studies, evaluating nine dressing types, were included. Ten direct treatment comparisons were made. Whilst there was increased healing associated with hydrogel and foam dressings compared with basic wound contact materials, these findings were based on data from small studies at unclear or high risk of bias. The mixed treatment comparison suggested that hydrocolloid-matrix dressings were associated with higher odds of ulcer healing than all other dressing types; there was a high degree of uncertainty around these estimates, which were deemed to be of very low quality.

**Conclusions/interpretation:**

These findings summarise all available trial evidence regarding the use of dressings to heal diabetic foot ulcers. More expensive dressings may offer no advantages in terms of healing than cheaper basic dressings. In addition, evidence pointing to a difference in favour of ‘advanced’ dressing types over basic wound contact materials is of low or very low quality.

**Electronic supplementary material:**

The online version of this article (doi:10.1007/s00125-012-2558-5) contains peer-reviewed but unedited supplementary material, which is available to authorised users.

## Background

Foot ulcers in people with diabetes are a common, serious and costly global health issue [1]. In 2007, the mean total reimbursement cost for a US Medicare patient with a diabetic foot ulcer was $33,000 (for all Medicare services) [2]. Dressings form a key part of ulcer treatment, with clinicians having many different types to choose from. Arguably, wound dressings are perceived as cheap and ‘inert’ items, thus consideration of their use in relation to existing evidence receives limited attention. However, as dressing types grow in number and complexity, and with claims of promoting healing, expenditure also increases. Drugs and devices prescribed in the UK National Health Service (NHS) are listed in the British National Formulary (BNF) [3] and the costs of non-hospital prescriptions in England are recorded by the NHS. Of the 201 BNF categories in 2010, the community prescription cost of the ‘wound management and other dressings’ section was £136 million [4], making it the 17th most costly section (the most costly section is ‘drugs for diabetes’ at over £700 million).

Dressings are widely used in wound care, with the aim of protecting and managing the wound and promoting healing. Several dressing types are available to treat complex wounds such as diabetic foot ulcers. We present a brief overview of dressing options using BNF-derived categories (electronic supplementary material [ESM] Table 1). Current guidelines for the treatment of diabetic foot ulcers maintain that clinical judgement should be used to select a moist wound dressing [5].

Nurses and podiatrists with whom we collaborate requested a review of current evidence regarding the use of dressings to heal diabetic foot ulcers in order to address treatment uncertainty in this area. Two recent systematic reviews have assessed the use of dressings to treat diabetic foot ulcers (literature searched to 2006 in the first and from 2006 to 2010 in the second) [6, 7] with the results of each search presented as a separate report with simple narrative review. The authors concluded that there was no evidence that any one dressing type was superior to another in terms of promoting ulcer healing. A further Cochrane systematic review that assessed the debridement of diabetic foot ulcers (literature searched to June 2009) found that significantly more hydrogel-treated ulcers healed compared with those treated with gauze or standard care (risk ratio 1.84 [95% CI 1.30, 2.61]) [8]. This estimate is based on three, poorly reported trials (follow-up period: 3 to 5 months) involving 198 participants.

To date, all previous reviews are limited by the lack of direct, head-to-head comparisons of alternative dressings in a field in which there are many dressing options. Thus, we aimed to evaluate the effects of alternative wound dressings on the healing of foot ulcers in people with diabetes by synthesising all available randomised controlled trial (RCT) evidence. As well as employing standard meta-analytical techniques for head-to-head comparisons we conducted a mixed treatment comparison (MTC) [9, 10], which allows consideration of both direct and indirect evidence to inform relative effectiveness estimates. These analyses were conducted from a Bayesian perspective; an alternative to the standard frequentist approach [11].

Furthermore, while there is growing interest in the conduct of MTC and the value of resulting data for clinical decision making, there is currently no established method of appraising and presenting the quality of MTC estimates. Quality assessment tools such as Grading of Recommendations Assessment, Development and Evaluation (GRADE) are available for standard pair-wise meta-analysis [12]; GRADE assesses the quality of estimates by evaluating: (1) the component studies; (2) how results compare across the different studies; and (3) the magnitude of the treatment effects from the synthesis. Such consideration is crucial in facilitating interpretation of results for clinical practice or policy. Without an equivalent process for MTC there is a risk that such estimates are used to inform decision making without due consideration of quality. We aimed to undertake a preliminary MTC quality assessment process in this study.

## Methods

### Study selection

This review was based on a pre-specified protocol [13]. We planned to include published or unpublished reports of RCTs, in any language and conducted in any country or setting, that evaluated the effects of wound dressings on the healing of diabetic foot ulcers. We accepted study authors’ definitions of what constituted a diabetic foot ulcer and included trials that recruited patients with any type of diabetic foot ulcer. There was no restriction in relation to participant age. We included any RCT in which the dressing type was the only systematic difference between treatment groups. Trials of non-dressing topical treatments (e.g. lotions, growth factors and skin replacements) were excluded as these were considered to be beyond the remit of this evidence synthesis; however trials of impregnated dressings and saline-moistened dressings (e.g. gauze) were eligible for inclusion. We also excluded trials that compared different brands of the same dressing type.

Our primary outcome was ulcer healing, measured using time to healing and/or number of ulcers completely healed within a specific time period (we assumed this period to be the trial follow-up time unless otherwise stated).

### Data sources and searches

The search string for CENTRAL (ESM text, section 1) was adapted for use in other databases, all being searched from inception to June 2011: Cochrane Wounds Group Specialised Register, Ovid MEDLINE, Ovid EMBASE and EBSCO CINAHL. The Ovid MEDLINE search was combined with the Cochrane Highly Sensitive Search Strategy for identifying randomised trials in MEDLINE: sensitivity- and precision-maximising version (2008 revision) [14]. The EMBASE and CINAHL searches were combined with the trial filters developed by the Scottish Intercollegiate Guidelines Network [15]. Reference lists of included studies and previous systematic reviews were also searched. We contacted appropriate manufacturing companies for details of any unpublished studies. Two review authors independently assessed the titles and abstracts of retrieved studies for relevance. After this initial assessment, we obtained all studies felt to be potentially relevant in full. We attempted to contact researchers to obtain any required additional information not contained in the trial reports.

#### Data extraction

Details of the eligible studies were extracted and summarised using a standardised data extraction sheet. Two review authors extracted data independently with disagreements resolved by discussion. If data were missing from reports, attempts were made to contact the study authors to obtain further information. Studies published in duplicate were included once but a comprehensive dataset was compiled from all publications.

##### Risk of bias assessment (individual studies)

Two review authors independently assessed each individual included study using the Cochrane Collaboration tool for assessing risk of bias, which addresses six specific domains [16]. Disagreements about risk of bias assessment were resolved by discussion and trial authors contacted where possible when data were missing. We classified trials as being at high risk of bias if they were rated 'No' for any of three key criteria (randomisation sequence, allocation concealment, and blinded outcome assessment).

### Data synthesis and analysis

A brief primer on this subject can be found in section 2 of the ESM text.

#### Relative treatment effects on ulcer healing: statistical analysis

##### Direct data

Where head-to-head (direct) treatment comparisons were reported in one trial only, ORs and 95% CIs were calculated. Where direct comparisons of dressings were available from more than one trial, appropriate standard meta-analyses (using ORs) were undertaken using Winbugs (available at www.mrc-bsu.cam.ac.uk/bugs). Results were reported with 95% credible intervals (CrIs)—the Bayesian equivalent of CIs, reflecting the uncertainty surrounding estimates. Unlike 95% CIs, 95% CrIs can be interpreted as: the (posterior) probability that these limits contain the parameter mean is 95%. Fixed and random effects models were considered and model fit assessed using the posterior mean of the residual deviance and the deviance information criterion (DIC).

##### Quality assessment of evidence generated using direct data

The overall quality of evidence surrounding estimates of effect using direct evidence only was assessed using GRADE [12]. GRADE assessment focuses not on individual studies but on a body of evidence and considers issues wider than threats to interval validity, including imprecision, inconsistency, indirectness and publication bias. Problems in any category lead to the quality of the evidence being decreased (we did not consider increasing the quality of evidence options). In reflecting the quality of an estimate drawn from multiple sources, GRADE aims to help the reader consider how confident we are that an effect estimate is correct [12]. Quality of evidence can be rated as high, moderate, low or very low.

#### Direct and indirect data: MTC

To maximise the use of all available trial data and to facilitate decision making regarding dressing choice we conducted an MTC [9, 10]. This approach links head-to-head comparison data from trials, via common comparators, into a network that can then be used to calculate indirect estimates of relative treatment effect. In a simple example where there are three treatments A, B and C compared in two head-to-head trials, A vs B and B vs C, as B is a common comparator the network A–B–C can be formed. These data can then be used to obtain an indirect estimate of the relative effects of A vs C. The MTC used OR as the measure of effectiveness and was conducted from a Bayesian perspective, again using Winbugs. Fixed and random effects models were fitted to these data with model fit assessed using residual deviance and DIC as before.

The treatment with the highest OR estimate in the MTC is expected to have the highest likelihood of healing diabetic foot ulcers. However, it is important to fully comprehend the uncertainty around such estimates. In addition to presenting CrIs, we represented uncertainty regarding treatment choice as the probability that each dressing was the ‘best’ treatment in terms of being the most likely to heal diabetic foot ulcers (when compared with all other evaluated treatments). To provide a complete overview of the spread of decision uncertainty around the choice of a ‘best’ treatment we then presented the probability of each treatment being the second best treatment and the third best and so on. Alternatively, this can be conceptualised, for each treatment, as a cumulative probability at each rank, summarised numerically as a surface under the cumulative ranking (SUCRA) for each treatment [17]. Thus, a SUCRA would be 1 (or 100%) when a treatment was certain to be the best and 0 (0%) when a treatment was certain to be the worst.

##### Inconsistencies

When direct and indirect evidence exists (i.e. a loop of evidence in the network diagram), inconsistencies between the ORs and intervals of these two sources may arise. We formally assessed for inconsistencies using the back calculation method suggested by Bucher et al [18, 19]. Briefly, where direct and indirect values could be compared, these values were calculated for each treatment, compared statistically against a null hypothesis that there would be no difference between them, and the *p* value for this test presented. We also extended the analysis to include an inconsistency model, which omitted consistency equations. Finally, potential inconsistencies between our direct and MTC estimates were also assessed by qualitatively comparing estimates of standard meta-analysis (direct) and MTC (direct and indirect). See section 3 of the ESM text for more information on inconsistencies.

##### Sensitivity analysis

We evaluated the sensitivity of the network to individual trials; where links were informed by more than one trial, we removed each trial one at a time (giving *n* − 1 for each analysis) and investigated the impact on the probability of which treatment was ‘best’.

##### Quality assessment of evidence generated from the MTC

We were also keen to reflect the quality of the evidence provided by the MTC so that this quality is transparently reflected in the strength of the conclusions made, as would be expected in other forms of evidence synthesis; however there is no established method for doing this in MTCs. We therefore modified the GRADE approach (we called this iGRADE) to allow us to access and communicate the quality of this MTC-derived evidence. We worked with the five categories in GRADE that allow the quality of evidence to be decreased; however we altered the focus of some categories so they were relevant for assessing an MTC (see ESM Table 2 for a full description of the iGRADE tool). No formal down-weighting of evidence was undertaken based on this assessment.

## Results

### Study characteristics

**Fig. 1:**
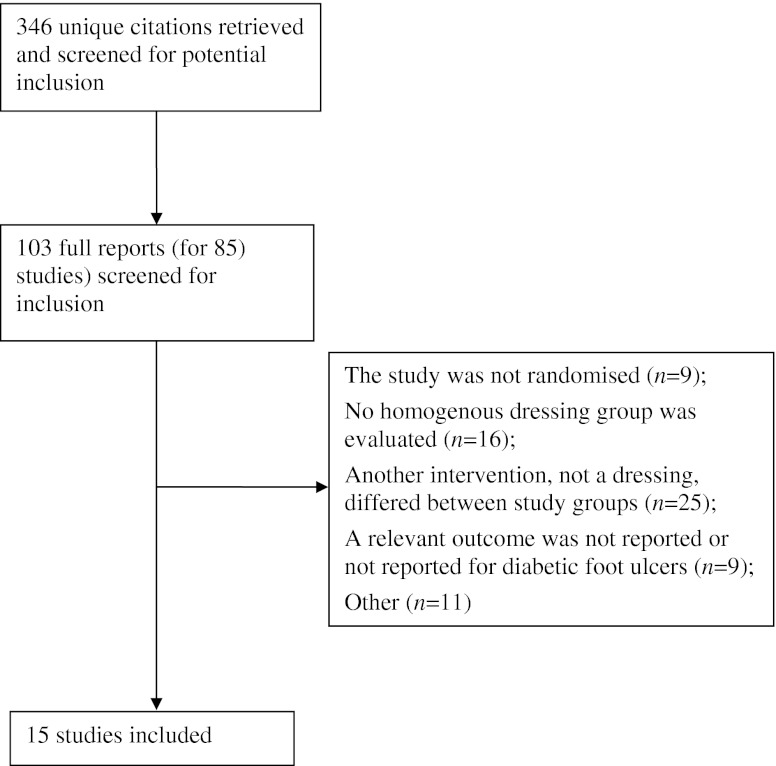
PRISMA overview of study identification and selection process

A Preferred Reporting Items for Systematic Reviews and Meta-Analyses (PRISMA) flowchart is presented in Fig. 1 (a summary of study characteristics for the 15 included studies is presented in ESM Table 3). All 15 included studies [20–34] reported the number of ulcers healed, while only three [21, 23, 25] reported median time to healing. Thus we focused our analyses on the proportion of ulcers healed. In terms of ulcer severity, four studies reported inclusion of Wagner grades 1 or 2 ulcers [22, 27, 28, 32] and one study specified that ulcers were superficial [23]. A further three studies specified that ulcers involving tendons, joint spaces and/or bone were excluded. Only one study specifically included more severe grade 3 and 4 ulcers [34]. Eight studies clearly excluded those with arterial disease [21, 23, 24, 26, 28, 30–32]. Eight studies excluded participants that had infected or sloughy ulcers [21, 23–27, 30, 32]. Only one study [33], comparing a basic contact wound with a hydrogel dressing_,_ clearly specified the inclusion of people with necrotic and infected wounds. The evidence base therefore overwhelmingly relates to people with less severe and less complex diabetic foot ulceration.

In terms of risk of bias, four included studies were deemed to be at high risk of bias [20, 23, 24, 33]. Only one study [26] was deemed at low risk of bias. The remaining ten studies were rated unclear for one or more key domains and hence we could not confidently judge their risk of bias (the outcome of the risk of bias assessment is summarised in ESM Table 3).

### Relative treatment effects on ulcer healing

#### Direct data

**Fig. 2:**
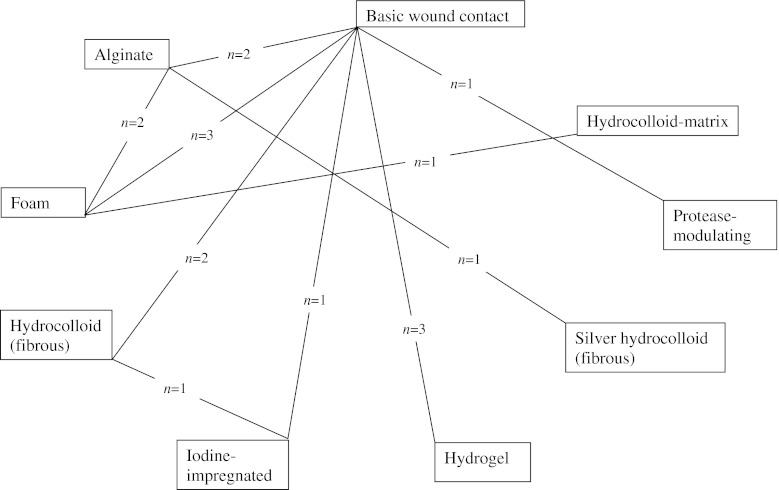
A network summary of all comparisons informed by direct trial data for wound dressings for diabetic foot ulcer healing. The lines link dressings that have been compared (in the treatment of diabetic foot ulcers) using a randomised controlled trial. (*n*=*x*) refers to the number of trials making this comparison. One three-arm trial was included that randomised to hydrocolloid (fibrous), iodine-impregnated and basic wound contact

A summary of the network of dressing trials that measured healing in participants with diabetic foot ulcers is illustrated in Fig. 2. Ten direct treatment comparisons were made in the 15 included trials; only five comparisons were informed by more than one trial where standard meta-analysis could be conducted (all fixed effect). The overall quality of evidence for each direct link was assessed using the GRADE quality of evidence scale (Table 1): four links were formed by low quality evidence and six by moderate quality evidence. It is important to note that three of these four links were informed by the same three-arm trial assessed as being at low risk of bias.

**Table 1:**
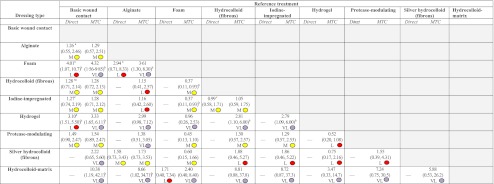
Results from direct and MTC analysis with assessment of overall quality of evidence using the GRADE (direct) and iGRADE (MTC) quality of evidence scales

The treatment on the horizontal axis is always the reference treatment

Direct data are presented as ORs and 95% CrIs for meta-analysed data (indicated by ^a^) and ORs and 95% CIs for non-pooled data (i.e. one trial). All MTC data are presented as ORs and 95% CrIs

In each cell, the left-hand value is the result of standard meta-analysis (using direct/head-to-head data only). The right-hand value is the MTC estimate (direct and/or indirect evidence).The shaded circles illustrate the assessed quality of evidence of estimates. Purple equates to very low quality evidence (VL); red equates to low evidence (L); yellow equates to moderate evidence (M) and green to high quality (H, none reported)

^a^Meta-analysed data

^b^Comparisons where credibility intervals do not cross 1

^c^Three-arm trial

Grey-shaded areas denote reverse odds ratios to those presented (with dressings in column as reference), which were not calculated

There was evidence that hydrogel dressings were associated with significantly higher odds of ulcer healing than basic wound contact dressings (OR 3.10 [95% CI 1.51, 5.50]) (Table 1). However, this finding was driven by low quality evidence, encompassing two small studies (sample sizes of 31 and 29 participants) [27, 33], one with unclear risk of bias and one at high risk of bias. Foam dressings were also associated with higher odds of ulcer healing compared with basic wound contract dressings (OR 4.01 [95% CI 1.07, 10.7]) (Table 1); again the estimate was considered to be of low quality. In the remaining five single study comparisons there was no evidence of any difference between one dressing and another. In general, estimates had large uncertainty due to low sample sizes.

#### MTC data

Based on assessment of fit, a fixed effect model was employed; there was minimal difference in mean residual deviances and DIC between the different models tested (fixed effect, simple random effects and full random effects, which accounted for correlation within the three-arm trial), thus the least complex model, given the limited data available for analysis, was applied.

There was a high degree of uncertainty in the many links in the network, especially those that were not informed by direct data (Table 1). Evidence remained that both foam and hydrogel dressings were expected to be associated with higher odds of ulcer healing than basic wound contact dressings, although uncertainty was high (Table 1). These dressings were estimated to be more effective than fibrous hydrocolloid and iodine-impregnated dressings, these results being driven by the more certain finding from a large, three-arm, trial that there was no difference in ulcer healing between fibrous hydrocolloid dressings and basic wound contract dressings and iodine-impregnated dressings and basic wound contact dressings. In this situation we must consider the quality of the evidence provided in these analyses (results of iGRADE analysis are presented in Table 1). In general, the network included several small studies leading to high imprecision; in addition, estimates were informed by studies with high risk of bias. We stress that the research deriving the estimates for fibrous hydrocolloid and iodine-impregnated dressings is regarded as being of higher quality, whereas evidence on hydrogel and foam dressings is classed as having more limited quality (Table 1) [22, 27, 29, 31, 33, 34].

A valuable feature of Bayesian methods is the ability to illustrate the impact of uncertainty on decision making by assessing the probability that each dressing treatment included in the network is the best in terms of ulcer healing. Notably, the treatment associated with the greatest probability of healing was hydrocolloid-matrix (70%, Table 2). This result reflects the high relative effect estimates generated by the MTC from available indirect evidence (OR 10.38 [95% CrI 1.19, 42.1], Table 1): hydrocolloid-matrix had higher odds of healing than foam and foam had higher odds of healing than basic wound contact. Again, interpretation of the evidence must also consider its quality; these results are drawn from low quality evidence and this limits the confidence we have in these conclusions. Estimates for the three dressings with the highest probability of being the best (hydrocolloid-matrix, hydrogel and foam) are informed by low quality evidence, while estimates for some dressings with 0% probability of being the best are informed by moderate quality evidence. SUCRA estimates reflect these findings considered cumulatively across the ranks 1–9: hydrocolloid-matrix dressings have a SUCRA of 92%, foam dressings 83% and hydrogel 78%, while basic wound contact has a SUCRA of 11% (Table 2), where a SUCRA of 100% means a treatment is certain to be the best and a treatment with a SUCRA of 0% is certain to be the worst.

**Table 2:**
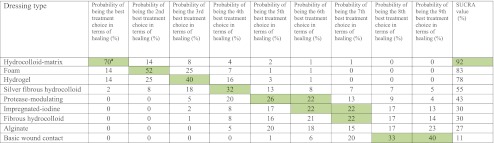
Treatment rankings

Table 2 shows the probability that each treatment is the best in terms of healing diabetic foot ulcers, then the second best in terms of healing diabetic foot ulcers and so on. The shaded square highlights the treatment with the highest probability in each column.

^a^Must be interpreted with caution owing to the risk of bias in the research

### Inconsistencies

There was one data loop where both direct and indirect data informed relative treatment effectiveness estimates; the possibility of inconsistency was investigated (ESM Fig. 1). While there was no evidence of statistically significant discrepancies between the direct and the indirect data, given the uncertainty in the data only very large differences were likely to result in statistical significance. Qualitative assessment was also undertaken (ESM text, section 4), which also concluded that there was no evidence of inconsistencies between direct and indirect data.

### Sensitivity analysis

The sensitivity of the network to specific studies was also investigated. In total, 11 analyses with 14 (rather than the total 15) included studies were performed and the probability of each dressing being the best was assessed. Basic wound contact layer, alginate, fibrous hydrocolloid, impregnated-iodine, and silver fibrous hydrocolloid dressings continued to have very low, or no probability of being the ‘best’ treatment in any sensitivity analysis. Hydrocolloid-matrix remained the most likely ‘best’ treatment in ten of the 11 analyses (probability of being ‘best’ ranging from 62% to 75%; ESM Table 4). The exception was when the largest study comparing hydrogel with a basic wound contact dressing was removed [34]. This resulted in the direct odds of healing with hydrogel (and the uncertainty around this estimate) increasing dramatically since the two remaining small trials both significantly favoured hydrogel with hydrogel having the highest probability of healing (62%, with hydrocolloid-matrix at 35%).

## Discussion

Currently, the findings from good quality research provide no evidence to suggest that fibrous hydrocolloid (hydrofibre), iodine-impregnated and protease-modulating dressings are more effective than basic wound contact materials in terms of ulcer healing. Thus, in the generally non-complex wounds studied, there was no evidence of a difference in healing between more expensive dressings compared with cheaper alternatives, nor between antimicrobial/antiseptic dressings and non-antimicrobial/antiseptic dressings.

Conversely, evidence pointing to a difference in favour of ‘advanced’ dressing types over basic wound contact materials is of low or very low quality. Thus for dressings such as hydrocolloid-matrix, we can only conclude that there is no high quality evidence to suggest that this dressing type is more effective than any other, or alternatively that any evidence suggesting that hydrocolloid-matrix dressings are more effective in healing diabetic foot ulcers is at high risk of bias.

### Strengths and limitations

The findings presented result from analysis of the most comprehensive evidence base available from across the world regarding the effect on healing of dressings to treat diabetic foot ulcers. While some may argue that the presence of sparse data should preclude any statistical synthesis of the evidence, we counter that clinicians cannot postpone treatment selection until high quality evidence has accumulated. Furthermore, comprehensive evidence synthesis highlights to researchers and clinicians the state of the current evidence base and its limitations as well as signposting where future research might focus. A further strength of this study is the application of an exploratory framework based on GRADE to undertake quality assessment of MTC estimates and the presentation of findings in light of this assessment.

We do acknowledge that there are limitations in synthesising sparse data, particularly in preventing further exploration of the potential impact of heterogeneity, for example length of trial follow-up. However, it is important to note that this is an issue for several evidence synthesis projects in wound care where data are sparse and follow-up times vary. In other networks, this has been dealt with by assuming a constant hazard of healing over time—although this assumption is potentially also not valid. In general, the limited evidence base prevented the application of a random effects model, however, we acknowledge that in other situations this type of model may be more appropriate than a fixed effects approach.

### Quality assessment of MTC estimates

We have developed and employed a preliminary framework for quality assessment of MTC evidence based on GRADE. In this example, we aimed to assess the feasibility and highlight potential challenges of applying quality assessment to MTC evidence. We note, however, that our modified approach has not been validated and is not recognised by GRADE.

It may be that other tools, rather than GRADE, would provide a better starting point for assessing the quality of MTC outputs. For example, GRADE’s remit extends to guiding clinical- and policy-level decision making, which are less relevant to MTC assessment. At the very least within our modified approach there are still several areas that need addressing, emphasising future challenges in developing such a scale: for example indirectness as a characteristic of the evidence is not easy to apply to MTC (as indirect data is a common feature that should not necessarily result in downgrading) and is perhaps more appropriately referred to as ‘inconsistency’ in this context. However, in standard GRADE, the term ‘inconsistency’ relates to unexplained heterogeneity. Within iGRADE we considered unexplained heterogeneity and inconsistency (the MTC-related meaning) together in one category. We then added a separate category that assessed the impact of sensitivity analysis on results. Our aim here was to assess the stability of the network and thus its estimates. Finally, imprecision could perhaps be omitted from this tool as it may be more useful for the reader to use their own judgement regarding the width of CrIs and what they mean. Quality assessment of MTC output is a complex area and further research is required; however, we emphasise that, currently, this work is the only example of formal quality assessment of MTC outputs.

## Conclusion and future research

These findings comprehensively summarise all trial evidence available to decision makers regarding the use of dressings to heal diabetic foot ulcers. This highlights that more expensive dressings may offer no advantages in terms of healing than cheaper, basic dressings. The work also highlights the risk of bias in some studies and how this can impact on interpretation of MTC findings.

The work provides a platform from which to consider future research. Given the large number of dressing options available to clinicians (while nine dressings have been evaluated here there are many more for which no RCT data exist), the design of any future studies should be driven by those questions regarded as high priority by decision makers and patients, and potentially guided by the data presented here. Finally, the analysis conducted here highlights that concerted efforts should be made in wound care, as in other fields, to utilise health professional and participant time only in the production of useful and valid research data.

## Electronic supplementary material

Below is the link to the electronic supplementary material.

ESM Table 1 PDF 10 kb

ESM Table 2 PDF 52 kb

ESM Table 3 PDF 81 kb

ESM Table 4 PDF 55 kb

ESM 1 PDF 126 kb

ESM Fig. 1 PDF 185 kb

## Abbreviations

BNF: British National Formulary
CrI: Credible interval
DIC: Deviance information criterion
GRADE: Grading of Recommendations Assessment, Development and Evaluation
MTC: Mixed treatment comparison
NHS: UK National Health Service
PRISMA: Preferred Reporting Items for Systematic Reviews and Meta-Analyses
RCT: Randomised controlled trial
SUCRA: Surface under the cumulative ranking
