# Supplementary material for: Systematic review and mixed treatment comparison: dressings to heal diabetic foot ulcers
Source: Diabetologia. 2012 Apr 29;55(7):1902–10. doi: 10.1007/s00125-012-2558-5 (PMC3369130; doi:10.1007/s00125-012-2558-5)
Supplement: Supplementary file 1 — PDF 10 kb [file 125_2012_2558_MOESM1_ESM.pdf]

Basic wound contact dressings	**Low-adherence dressings and wound contact materials:** usually cotton pads which are placed directly in contact with the wound. They can be either non-medicated (e.g. paraffin gauze dressing) or medicated (e.g. containing povidone iodine or chlorhexidine).<br><br>**Absorbent dressings:** applied directly to the wound and may be used as secondary absorbent layers in the management of heavily exuding wounds.
Advanced wound dressings	**Alginate dressings:** highly absorbent and come in the form of calcium alginate or calcium sodium alginate and can be combined with collagen. The alginate forms a gel when in contact with the wound surface which can be lifted off with dressing removal or rinsed away with sterile saline.<br><br>**Hydrogel dressings:** consist of a starch polymer and up to 96% water. These dressings can absorb wound exudate or rehydrate a wound depending on the wound moisture levels. They are supplied in either flat sheets, an amorphous hydrogel or as beads·<br><br>**Films - permeable film and membrane dressings:** permeable to water vapour and oxygen but not to water or microorganisms·<br><br>**Soft polymer dressings:** dressings composed of a soft silicone polymer held in a non-adherent layer. They are moderately absorbent.<br><br>**Hydrocolloid dressings:** usually composed of an absorbent hydrocolloid matrix on a vapour-permeable film or foam backing.<br><br>**Fibrous hydrocolloid dressing:** Fibrous hydrocolloids have been developed which resemble alginates and are not occlusive.<br><br>**Hydrocolloid-matrix dressing:** Described in the BNF as a polyurethane matrix with absorbent particles and waterproof polyurethane film.<br><br>**Foam dressings:** contain hydrophilic polyurethane foam and are designed to absorb wound exudate and maintain moist wound surface.<br><br>**Capillary-action dressings:** consist of an absorbent core of hydrophilic fibres held between two low-adherent contact layers.<br><br>**Odour-absorbent dressings:** dressings that contain charcoal and are used to absorb wound odour. Can be used in conjunction with a secondary dressing to improve absorbency.
Anti-microbial dressings	**Iodine-impregnated dressings:** release free iodine when exposed to wound exudate, which is thought to act as a wound antiseptic.<br><br>**Silver-impregnated dressings:** used to treat infected wounds as silver ions are thought to have antimicrobial properties. Silver versions of most dressing types are available (e.g. silver foam, silver hydrocolloid etc).<br><br>**Other antimicrobial dressings:** these dressings are composed of a gauze or low-adherent dressing impregnated with an ointment thought to have antimicrobial properties.
Specialist dressings	**Protease-modulating matrix dressings**: are proposed to alter the activity of proteolytic enzymes in chronic wounds·

ESM Table 1 Overview of common wound dressings based on British National Formulary categories.[1]

1. British Medical Association and Royal Pharmaceutical Society of Great Britain (2010). British National Formulary, Sept 2010;60, Appendix 8:Wound management Products and Elastic Hosiery. Available from bnf.org.uk/bnf/bnf/current/104946.htm, accessed
