# Supplementary material for: Systematic review and mixed treatment comparison: dressings to heal diabetic foot ulcers
Source: Diabetologia. 2012 Apr 29;55(7):1902–10. doi: 10.1007/s00125-012-2558-5 (PMC3369130; doi:10.1007/s00125-012-2558-5)
Supplement: Supplementary file 2 — PDF 52 kb [file 125_2012_2558_MOESM2_ESM.pdf]

GRADE CATEGORY	GRADE Definition and guidance	iGRADE CATEGORY	iGRADE Definitions and guidance	iGRADE ISSUES
**Limitations in design**	**Risk of Bias**<br><br>• **If you think any limitations were negligible choose <u>no</u>**<br>• **If you think there were serious limitations choose <u>serious</u>**<br>• **If you think there were very serious limitations choose <u>very serious</u>**	**Limitations in design**	**Use GRADE limitations in design rating for DIRECT links to assess the MTC estimates these links clearly contributed to.**<br><br>**No:** GRADE *limitations in design* category recorded as 'no' for all links identified as informing the MTC estimate.<br>**Serious:** GRADE *limitations in design* category recorded as serious for one or more links identified as informing the MTC estimate, but none identified as very serious.<br>**Very serious:** GRADE *limitations in design* category recorded as very serious for one or more links identified as informing the MTC estimate.	**Qualitative assessment of risk of bias difficult for indirect evidence. When direct and indirect evidence are available, this assessment may be subjective.**
**Inconsistency**	**Unexplained heterogeneity of results**<br><br>• **If you think any inconsistency was negligible choose <u>no</u>**<br>• **If you think there was serious inconsistency choose <u>serious</u>**<br>• **If you think there was very serious inconsistency choose <u>very serious</u>**	**Sensitivity of results**	**Judgement based on the impact of sensitivity analysis on the MTC network and thus estimates (e.g. removing each trial where there are two or more informing a link, or sensitivity to alternative priors in random effects analysis)**<br><br>**No:** No or small change in estimate and intervals<br>**Serious:** Some notable change in estimate and intervals<br>**Very serious:** Large change in estimate and intervals	**Does not address unexplained heterogeneity per se**
**Indirectness**	**Indirect comparison**<br><br>• **If you think the evidence is direct choose <u>no</u>**	**Indirectness/Inconsistency**<br>Within GRADE the term inconsistency is used to refer to unexplained heterogeneity. Within MTC inconsistency has	**Define the type of data available for each MTC comparison as follows:**<br>1. Direct or indirect only: *No heterogeneity*<br>2. Direct, indirect or mixed (direct and indirect): *heterogeneity*	**Assessment of heterogeneity based in DIRECT links is challenging**

	• **If you have serious doubts about directness choose <u>serious</u>**<br>• **If you have very serious doubts about directness choose <u>very serious</u>**	meaning specific to agreement between direct and indirect data. Furthermore, in GRADE the presence of indirectness is taken as a reason to downgrade evidence – however in the context of an MTC where indirect data is expected and ideally adds value such an approach does not make sense. Thus we merged these categories resulting in joint assessment of unexplained heterogeneity and/or assessment of inconsistency where possible.	*3.* Mixed: *No heterogeneity: statistical inconsistencies*<br>4. Mixed: *No heterogeneity; No statistical inconsistencies*<br><br>**No:** 1 and 4<br>**Serious:** 2, 3<br>**Very serious:** n/a	**Cannot always assess for inconsistencies**
**Imprecision**	**CIs around estimates of treatment effect**<br><br>• **If you think the results were precise choose <u>no</u>**<br>• **If there was serious imprecision choose <u>serious</u>**<br>• **If there was very serious imprecision choose <u>very serious</u>**	**Imprecision**	Judged by the size of CrI around ORs. As ORs were used to analyse data with relative high number of events a more conservative interval width used than would have been employed were data presented using risk ratios.<br><br>**No:** uncertainty judged to be reasonable (upper interval < 2·5)<br>**Serious:** judged to be inadequate (upper interval > 2·5<5)<br>**Very serious:** (upper interval > 5)	
**Publication bias**	• **If you think there is no evidence of publication bias choose <u>unlikely</u>**<br>• **If there is high probability of publication bias choose <u>likely</u>**<br>• **If there is very high probability of publication bias choose**	**Publication bias**	**Use GRADE limitations in design rating for DIRECT links to assess the MTC estimates these links clearly contributed to.**<br><br>**Unlikely:** Grade *publication bias* category recorded as unlikely for links identified as informing the MTC estimate.<br>**Likely:** Grade *publication bias* category recorded as likely for one or more links identified as informing the MTC estimate and	**Qualitative assessment of publication bias difficult for indirect evidence**<br><br>Again, in the presence of both direct and indirect evidence there is the need to consider potential publication

	**<u>very likely</u>**		none identified as very likely.<br>**Very likely:** for GRADE *publication bias* category recorded as very likely for one or more link identified as informing the MTC estimate.	bias in the indirect links as well as the direct links informing the same comparison. Yet, outlined in the discussion of limitations, assessing potential bias in indirect comparison is complex. If, for example, AC is biased (missing studies) favouring A and BC is biased (missing studies) favouring B, then the AB indirect estimate will be unbiased if the bias in AC is similar to the bias in BC.

ESM Table 2 Quality assessments of mixed treatment comparison estimates using iGRADE: comparison with the GRADE tool.
