# Supplementary material for: Systematic review and mixed treatment comparison: dressings to heal diabetic foot ulcers
Source: Diabetologia. 2012 Apr 29;55(7):1902–10. doi: 10.1007/s00125-012-2558-5 (PMC3369130; doi:10.1007/s00125-012-2558-5)
Supplement: Supplementary file 3 — PDF 81 kb [file 125_2012_2558_MOESM3_ESM.pdf]

ESM Table 3

Study	Duration of follow-up (weeks)	Interventions evaluated (No. participants)	Number healed (%)[b]	Inclusion criteria	Mean Age in years (SD where reported, otherwise range where reported)	Mean Ulcer duration in days (SD where reported), unless stated·	Mean Ulcer size in cm^2 (SD where reported) unless stated	Risk of Bias assessment (1) Generation of randomisation sequence (2) Allocation concealment (3) Blinded outcome assessment	Funding
Ahroni 1993	4 weeks	Dry gauze (19)<br>Alginate (20)	7 (37)<br>5 (25)	Patients with diabetic foot ulcers that penetrated the epidermis but did not significantly involve joint spaces, tendons or bone·	65·4 (9·3)<br>61·2 (11·0)	74·9 (130·4)<br>132·9 (320·6)	166·7 mm^2 (211·1)<br>193·2 mm^2 (346·4)	(1) Unclear<br>(2) Unclear<br>(3) High risk	Commercial
Donaghue 1998	8 weeks	Saline-moistened gauze (25)<br>Alginate (50)	9 (36)<br>24 (48)	Diabetic patients who were treated for foot ulceration of at least 21 years of age, adequate nutritional intake, as indicated by a serum albumin of > 2·5 grams/dl; adequate blood flow to the lower extremities, as indicated by palpable pulse and/or normal non-invasive tests; and foot ulceration of at least 1 cm^2 in size after initial debridement. No evidence of osteomyelitis, or clinical signs of infection	60 (33-79)<br>59 (30-81)	225 (104)<br>146 (73)	2·99 (0·62)<br>2·60 (0·50)	(1) Unclear<br>(2) Unclear<br>(3) High risk	Commercial
Blackman 1994	8 weeks	Wet-to-dry saline gauze (7)<br>Foam (11)	0·5 (0)[a]<br>3·5 (27)	Diabetic patients (type 1 and 2) with foot ulcers free of hard eschar. Wagner Grade: 1 or 2	51 (4·0)<br>59 (5·0)	28·6<br>25. 7	1·81 (0·75)<br>2·67 (1·20)	(1) Unclear<br>(2) Unclear<br>(3) Unclear	Commercial
Mazzone 1993	8 weeks	Wet-to-dry saline gauze dressing (8)<br>Foam (11)	2 (25)<br>7 (64)	Diabetic foot ulcer	Not reported	Not reported	Not reported	(1) Unclear<br>(2) Unclear<br>(3) Unclear	Commercial
Roberts 2001	13 weeks	Saline-soaked low adherent (16)<br>Foam (14)	4 (25)<br>6 (43)	Type 1 diabetes with neuropathic ulcer. ABPI >0·8	Not reported	Not reported	Median = 144·5mm^2<br>Median = 114·5mm^2	(1) Unclear<br>(2) Unclear<br>(3) Unclear	Commercial

Jeffcoate 2009	24 weeks	Low adherence (106)<br>Hydrocolloid (fibrous) (103)<br>Iodine-impregnated (108)	41 (39)<br>46 (45)<br>48 (44)	Patients with type 1 or 2 diabetes, aged 18 years or more having a foot ulcer present for at least 6 weeks. Ulcer cross-sectional area of between 25 and 2500 mm^2. Those with an ankle brachial pressure index of more than 0·7 or toe systolic pressure more than 30 mmHg·<br>No ulcer on either foot extending to tendon, periosteum or bone. No infection of the bone or soft tissue infection requiring treatment with systemic antibiotics.	61·9 (12·8)<br>58·8 (13·2)<br>59·5 (11·5)		25-100mm^2: 50; 53; 48<br>101-250mm^2 :34; 36; 34<br>2501-2500mm^2: 22;16; 24<br><br>*Data categorised into three groups: (1) 25-100 mm2; (2) range 101-250 mm2 and (3) range 251-2500 mm2. Reported as n(%) per category per group.*	(1) Low risk<br>(2) Low risk<br>(3) Low risk	Non-commercial
Piaggesi 2001	(Max 350 days)	Saline-moistened gauze (10)<br>Hydrocolloid (fibrous) (10)	10 (100)<br>9 (90)	Diabetic patients (type 1 or type 2) for over 5 years, between age 18 to 75 years, foot ulcer more than 3 weeks, >1 cm wide and 1 cm deep, good peripheral blood supply (palpable peripheral pulses or ABPI >0·9). No active infection·	61·3 (7·5)<br>63·1(4·6)	5·9 (1·3) weeks<br>6·8 (2·6) weeks	Ulcer vol : 19·2 (6·4) cm^3<br>Ulcer vol : 22·6 (8·4) cm^3	(1) Low risk<br>(2) Unclear<br>(3) Unclear	Non-commercial
Jensen 1998	16 weeks	Saline-moistened gauze (17)<br>Hydrogel (14)	6 (35)<br>11 (79)	No signs of infection in the ulcer or the peri-wound tissue. Diabetic foot ulcer measuring at least 1cm diameter, Diabetic patients with foot ulcers of Wagner grade 2 defined as full thickness into subcutaneous tissue but not involving tendon, joint capsule or bone, having palpable pulse and willingness to comply to the treatment·	Not reported	Not reported	Not reported	(1) Unclear<br>(2) Unclear<br>(3) Unclear	Commercial
Vandeputte 1997	12 weeks	Dry gauze (14)<br>Hydrogel (15)	7 (50)<br>14 (93)	Patients with diabetes and a wound on the foot. Necrotic and/or infected wound were included. Only patients receiving systemic antibiotics were excluded from the trial·	65·3 (14·3)<br>62·6 (14·7)	Not reported	Not reported	(1) Unclear<br>(2) Unclear<br>(3) High risk	Not reported

D'Hemecourt 1998	20 weeks	Wet-to-moist saline gauze (68)<br>Hydrogel (70)	15 (22)<br>25 (36)	Patients of 19 years of age or older with type 1 or type 2 diabetes mellitus with at least one full thickness (stage 3 or 4), chronic diabetic foot ulcer present for at least 8 weeks prior to the study. A target area between 1·0 and 10 cm^2 was required. Ulcers with osteomyelitis affecting the area of the target ulcer were excluded.	Not reported	42·0 (42·0) weeks*<br>52·8 (60·9) weeks*<br>*n=24 only	3·5 (3·53)<br>3·2 (2·75)	(1) Unclear<br>(2) Unclear<br>(3) Low risk	Not reported
Veves 2002	12 weeks	Saline-moistened gauze (138)<br>Protease-matrix (138)	39 (28)<br>51 (37)	Diabetic patients above 18 years of age with foot ulcers of at least 30 days duration, Wagner grade 1 or 2 and area of at least 1 cm^2. Patients had adequate circulation wound that was debrided of necrotic tissue at enrolment. No clinical signs of infection or a target wound that had exposed bone.	59 (37-83)<br>58 (23-85)	Median;(range): 3 (1–144)<br>Median;(range): 3 (1–84)	3·1 (0·1–42·4)<br>2·5 (0·2–27·4)	(1) Unclear<br>(2) Unclear<br>(3) Unclear	Commercial
Baker 1993	12 weeks	Alginate (10)<br>Foam (10)	4 (40)<br>9 (90)	Patients above 18 years of age with clean diabetic foot ulcers that were neuropathic in origin located on weight bearing areas of the foot. No necrotic, sloughy ulcers or peripheral vascular disease, no presence of infection in ulcerative foot·	54·1 (15·8)<br>58·9 (18·5)	26·3 (49·2)<br>19·8 (21·9)	0·82 (0·73)<br>0·89 (0·62)	(1) Low risk<br>(2) Unclear<br>(3) Unclear	Not reported
Foster 1994	8 weeks	Alginate (15)<br>Foam (15)	8 (53)<br>9 (60)	Patients above the age of 18 years with clean diabetic foot ulcers and were willing and able to comply with study protocol and without sloughy, necrotic or infected ulcer·	70<br>61	170<br>107	79 mm^2<br>88 mm^2	(1) Unclear<br>(2) Unclear<br>(3) Unclear	Not reported

Jude 2007	8 weeks	Alginate (67)<br>Silver hydrocolloid (fibrous) (67)	15 (22)<br>21 (31)	Patients with type 1 or type 2 diabetes mellitus (HbA1c ≤ 12%), serum creatinine ≤ 200 mol/l diabetic foot ulcers classed as Wagner grade 1 or 2 and of neuropathic or neuro-ischaemic aetiology. All wounds > 1cm^2 in area. Adequate arterial perfusion as defined by ankle-to-brachial index > 0·8, great toe systolic blood pressure > 40 mmHg or forefoot TcPO2 > 30 mmHg	58·9 (12·6)<br>61·1 (11·4)	1·2 (2·1) years<br>1·4 (2·6) years	3·1 (4·1)<br>4·2 (7·8)	(1) Low risk<br>(2) Unclear<br>(3) Unclear	Commercial
Clever 1995	16 weeks	Foam (20)<br>Hydrocolloid-matrix (20)	14 (70)<br>16 (80)	Patients aged 18 to 80 years with a pure neuropathic superficial ulcer 1 to 5 cm in diameter and with no clinical and radiological signs of osteomyelitis or tendon involvement·	53·2 (14·6)<br>58·9 (11·64)	165 (318·68)<br>162·37 (325·55)	207·83 mm^2<br>205·09 mm^2	(1) Unclear<br>(2) Unclear<br>(3) High risk	Commercial

ESM Table 3: Summary of trial characteristics of included studies.
[a]0·5 added to group and healed values for this study to allow calculation of odds ratio.
[b]where proportion of ulcers healed data were presented we assumed that when randomised participants were not included in an analysis, their wound did not heal (that is they were considered in the denominator but not the numerator). Ankle brachial pressure index (ABPI). Standard deviation (SD).
