# Supplementary material for: Systematic review and mixed treatment comparison: dressings to heal diabetic foot ulcers
Source: Diabetologia. 2012 Apr 29;55(7):1902–10. doi: 10.1007/s00125-012-2558-5 (PMC3369130; doi:10.1007/s00125-012-2558-5)
Supplement: Supplementary file 4 — PDF 55 kb [file 125_2012_2558_MOESM4_ESM.pdf]

	**Probability of being the best treatment choice in terms of healing (%)**										
	**Ahroni 1993 removed**	**Donaghue 1998 removed**	**Blackman 1994 removed**	**Mazzone 1993 removed**	**Roberts 2001 removed**	**Piaggesi 2001 removed**	**D'Hemecourt 1998 removed**	**Vandeputte 1997 removed**	**Jenson 1998 removed**	**Baker 1993 removed**	**Foster 1994 removed**
Matrix hydrocolloid	71	66	67	67	72	70	35	73	72	63	75
Hydrogel	9	21	17	18	10	14	62	8	9	21	6
Foam	14	11	12	12	16	13	2	14	15	9	18
Silver fibrous hydrocolloid	6	2	3	3	2	3	1	3	3	7	1
Basic wound contact layer	0	0	0	0	0	0	0	0	0	0	0
Alginate	0	0	0	0	0	0	0	0	0	0	0
Fibrous hydrocolloid	0	0	0	0	0	0	0	0	0	0	0
Impregnated-iodine	0	0	0	0	0	0	0	0	0	0	0
Protease modulating	0	0	0	0	0	0	0	0	0	0	0

ESM Table 4 Sensitivity analyses. Table shows the probability that each treatment is the best in terms of healing diabetic foot ulcers when one trial (named in column) had been removed. Shaded cell highlights study with highest probability for each column.
