# Supplementary material for: Systematic review and mixed treatment comparison: dressings to heal diabetic foot ulcers
Source: Diabetologia. 2012 Apr 29;55(7):1902–10. doi: 10.1007/s00125-012-2558-5 (PMC3369130; doi:10.1007/s00125-012-2558-5)
Supplement: Supplementary file 5 — PDF 126 kb [file 125_2012_2558_MOESM5_ESM.pdf]

ESM Section 1 We searched the Cochrane Central Register of Controlled Trials (known as CENTRAL) using the following exploded MeSH headings and keywords:

#1 MeSH descriptor Occlusive Dressings explode all trees
#2 MeSH descriptor Biological Dressings explode all trees
#3 MeSH descriptor Alginates explode all trees
#4 MeSH descriptor Hydrogels explode all trees
#5 MeSH descriptor Silver explode all trees
#6 MeSH descriptor Honey explode all trees
#7 (dressing* or alginate* or hydrogel* or "foam" or "bead" or "film" or "films" or tulle or gauze or non-adherent or "non adherent" or silver or honey or matrix):ti,ab,kw
#8 (#1 OR #2 OR #3 OR #4 OR #5 OR #6 OR #7)
#9 MeSH descriptor Foot Ulcer explode all trees
#10 MeSH descriptor Diabetic Foot explode all trees
#11 diabet* NEAR/3 ulcer*:ti,ab,kw
#12 diabet* NEAR/3 (foot or feet):ti,ab,kw
#13 diabet* NEAR/3 wound*:ti,ab,kw
#14 (#9 OR #10 OR #11 OR #12 OR #13)
#15 (#8 AND #14)

ESM Section 2 Brief Primer

We start by assuming there is a medical condition for which treatments A, B, C and D are all separate possible treatment options for the same type of patients with all treatments aiming to achieve the same outcome.

In terms of evidence there are five separate trials (direct data) that compare these interventions in the patient group of interest and for the outcome of interest. Thus, when making treatment decisions health professionals need to make a choice in part informed by the effectiveness evidence provided by these trials. The outcome of interest is dichotomous (i.e. healed vs. not healed).

Trial 1 = A vs. B
Trial 2 = A vs. B
Trial 3 = C vs. D
Trial 4 = C vs. D
Trial 5 = B vs. D

These data could potentially lead to two meta-analyses reported with ORs and CI ir CrIs as is common in systematic reviews such as Cochrane reviews.

Meta-analysis 1: A vs. B (n=2: trials 1 and 2)
Meta-analysis 2: C vs. D (n=2: trials 3 and 4)

And the results of a single trial

B vs. D (n = 1: trial 5)

However, from a decision making perspective as well as knowing the relative effects from direct data:
A vs B
C vs D
B vs D

it would be useful to know the relative effects of the other potential comparisons, thus increasing insight into which of the four treatment is potentially the most effective:
A vs C
A vs D
B vs C

Whilst there are no pair-wise trials of A vs. C, A vs. D and B vs. C there is an analytical technique, commonly referred to as mixed treatment comparison (MTC) or network meta-analysis, which can estimate these indirect treatment effects using the existing pair-wise data. Importantly, these techniques allow randomisation to be preserved; however, they do require certain assumptions to be made — a key assumption being that the data obtained from each trial can be considered similar to the data that would have been obtained had one trial with four arms evaluating all treatments simultaneously been conducted.

To understand the indirect estimates that can be obtained, existing trials are displayed as a network — simply a way of understanding how data are linked. In this example the network is shown below that is formed of existing trial evidence (black lines) and indirect data that can be obtained (red lines).

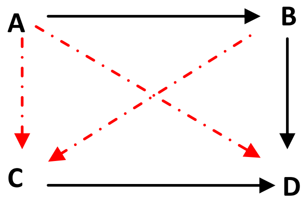

The additional value of mixed treatment comparison techniques is that whilst an extension and application of meta-analysis, the approach can be used to present more intuitive summarises of multiple comparisons from a decision making perspective. Thus the final output of a MTC can be presented as the probability that each treatment the ‘best’ in terms of the outcome that is the focus of the analysis.

Thus from starting with four possible treatment options a MTC has the potential to highlight which may be the best and importantly illustrated how certain we are about this decision. Importantly however current methods do not enable these analyses to consider the *quality* of the evidence derived from MTC.

ESM Section 3 Why assess inconsistencies?

The MTC approach used here (aggregating relative treatment effects) is appealing to decision-makers as it incorporates indirect and direct evidence, whilst maintaining the randomisation of each included study. However, as with standard meta-analysis, synthesising data in a MTC implicitly assumes that the relative treatment effects being estimated in different trials are consistent. Consistency means that (for a fixed effects model) all networked trials would be expected to estimate the same relative treatment effect for a treatment, had that treatment been included in the trial. In essence, this assumption relies on there being no important differences between trials (e.g. characteristics of patient populations) that would impact on relative treatment effects. In MTC there is particular focus on consistency between direct and indirect data: for a full overview of this issue see Dias 2010·[1]

1. Dias S, Welton NJ, Caldwell DM, Ades AE. Checking consistency in mixed treatment comparison meta-analysis. *Stat Med* 2010;**29**:932-944.

ESM Section 4 What does qualitative assessment of direct and indirect evidence suggest about consistency?

Assessing the data qualitatively we noted that only one link, that between basic wound contact and foam dressings, had an indirect point estimate that differed considerably from the direct data (ESM Figure 1), this was likely driven by the high and uncertain estimates in two of the three studies that contributed direct evidence· For the basic wound contact—alginate link, whilst the direct and indirect estimates were close, the indirect estimate was much more uncertain. Interestingly, this link was the only one to have conflicting direct evidence· The direct and indirect estimates for the alginate—foam were close, with the indirect link having slightly more uncertainty. The direct evidence in this link had very differently sized estimates, although this might be explained by the large uncertainty in one study which had only 20 participants.
