# Supplementary material for: Systematic review and mixed treatment comparison: dressings to heal diabetic foot ulcers
Source: Diabetologia. 2012 Apr 29;55(7):1902–10. doi: 10.1007/s00125-012-2558-5 (PMC3369130; doi:10.1007/s00125-012-2558-5)

ESM Fig. 1 Assessment of inconsistencies in data loop. From top to bottom for each link: Study ORs, results from standard fixed effects meta-analysis, results from MTC, results when relative effect estimated with only indirect data (direct data removed), results of statistical analysis of direct versus indirect evidence data. * ***Median rather than mean values have been presented for indirect data.***

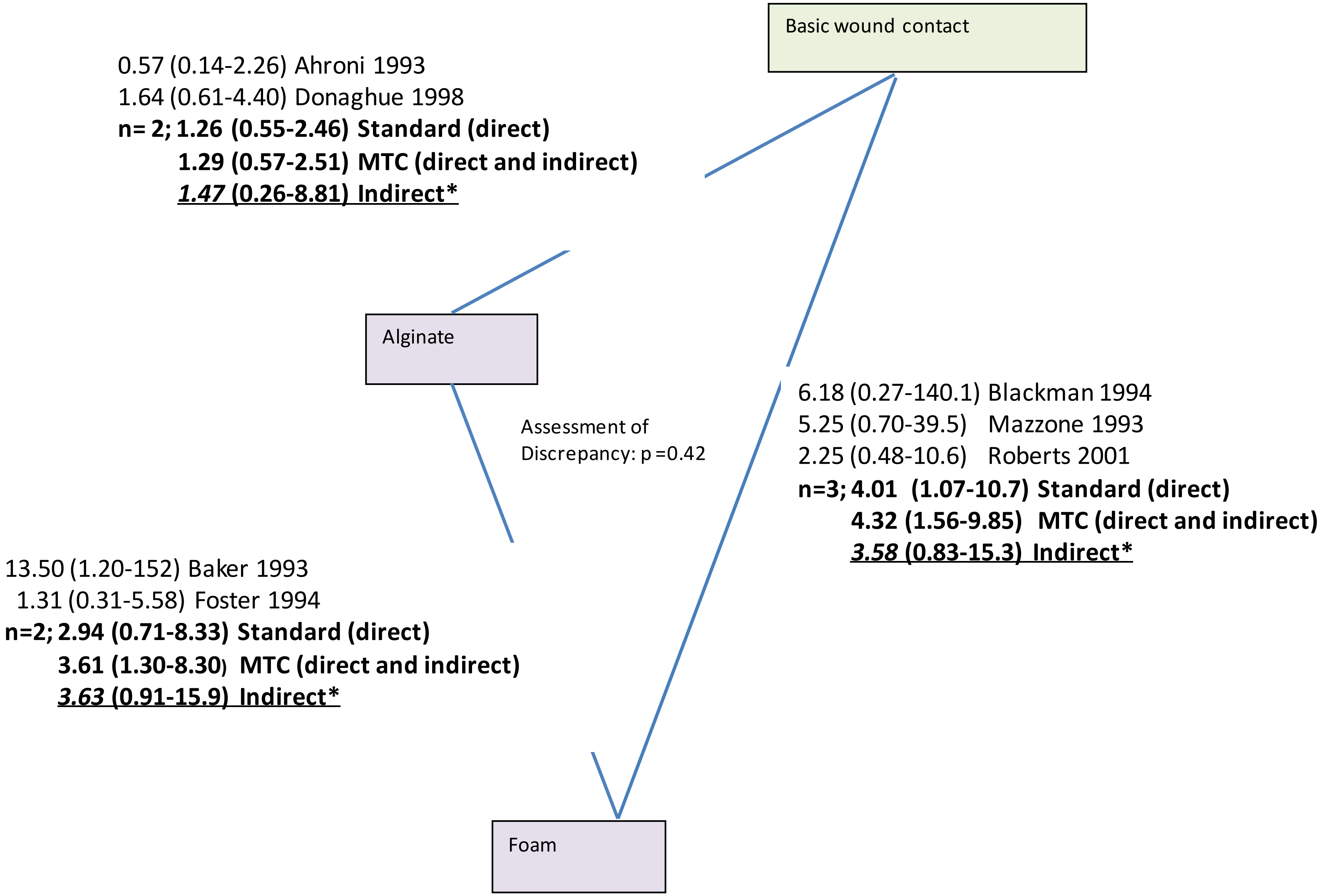

Supplement: Supplementary file 6 — PDF 185 kb [file 125_2012_2558_MOESM6_ESM.pdf]
